# Global Review on *Naegleria fowleri* Cases: Contemporary Epidemiology, Diagnosis, Treatment and Outcomes

**DOI:** 10.3390/idr18020025

**Published:** 2026-03-24

**Authors:** Andreas Sarantopoulos, Annalisa Quattrocchi, Ioannis Kopsidas, Oliver A. Cornely, Danila Seidel, Itamar Grotto, Zoi Dorothea Pana

**Affiliations:** 1 Medical School, European University of Cyprus (EUC), 2404 Nicosia, Cyprus; as192173@students.euc.ac.cy; 2 Department of Primary Care and Population Health, Medical School, University of Nicosia (UNIC), 2408 Nicosia, Cyprus; quattrocchi.a@unic.ac.cy; 3 Center for Clinical Epidemiology and Outcome Research (CLEO), 15451 Athens, Greece; 4 Cologne Excellence Cluster on Cellular Stress Responses in Aging-Associated Diseases (CECAD), Institute of Translational Research, Faculty of Medicine and University Hospital Cologne, University of Cologne, 50937 Cologne, Germany; oliver.cornely@uk-koeln.de (O.A.C.); danila.seidel@uk-koeln.de (D.S.); 5 Excellence Center for Medical Mycology (ECMM) and Center for Integrated Oncology Aachen Bonn Cologne Duesseldorf (CIO ABCD), Division of Infectious Diseases, Department I of Internal Medicine, Faculty of Medicine and University Hospital Cologne, University of Cologne, 50937 Cologne, Germany; 6 German Centre for Infection Research (DZIF), Partner Site Bonn-Cologne, 38124 Cologne, Germany; 7 Clinical Trials Centre Cologne (ZKS Köln), Faculty of Medicine and University Hospital Cologne, University of Cologne, 50931 Cologne, Germany; 8 Department of Epidemiology, Biostatistics and Community Health Sciences, Ben-Gurion University (BGU), Beer-Sheva 8410501, Israel; itamar.grotto@gmail.com; 9 Department of Basic & Clinical Sciences, Medical School Block C, 21 Ilia Papakyriakou, 2414 Engomi, P.O. Box 24005, CY-1700 Nicosia, Cyprus

**Keywords:** *Naegleria fowleri*, primary amoebic meningoencephalitis, PAM, free-living amoeba, freshwater exposure, epidemiology, polymerase chain reaction (PCR), amphotericin B, miltefosine

## Abstract

Background/Objectives: Primary amoebic meningoencephalitis (PAM) is a rare, fulminant, and often fatal central nervous system infection caused by the opportunistic free-living amoeba *Naegleria fowleri*. Although *Naegleria* species are widely present in freshwater and soil worldwide, human disease is associated specifically with pathogenic *N. fowleri* rather than the many nonpathogenic environmental species, and virulence may vary across *N. fowleri* isolates. This systematic review aimed to synthesize contemporary global data from 2000 to 2024 to identify recent trends in epidemiology, clinical presentation, diagnosis, treatment, and outcomes. Methods: A systematic literature search was conducted across PubMed, Scopus, and the Cochrane Library, identifying 58 eligible publications encompassing 66 individual cases. Results: Most reports originated from the United States, India, and China. The median patient age was 14 years, with 78% of cases occurring in males. Annual case reports increased from one per year (2000–2005) to over four per year (2020–2024), reflecting either a true rise in incidence or improved detection. Common presenting symptoms included fever, headache, and altered mental status. Diagnosis was confirmed via polymerase chain reaction (PCR) testing or post-mortem biopsy in nearly one-third of cases. Treatment regimens varied, with amphotericin B and miltefosine being the most frequently used agents. Overall mortality was 83%, with survival strongly associated with early initiation of combination therapy. Pediatric patients had a higher survival rate (22%) compared to adults (7.1%). Conclusions: The findings highlight the need for heightened clinical awareness, especially in the context of climate-driven ecological changes that may expand *N. fowleri*’s geographic range. This review underscores critical gaps in surveillance and diagnostics and emphasizes the importance of a One Health approach to addressing emerging threats like PAM. Further research into novel therapeutics, rapid diagnostics, and global case reporting systems is urgently needed.

## 1. Introduction

*Naegleria fowleri*, a free-living thermophilic amoeba, is the etiological agent of primary amoebic meningoencephalitis (PAM)—a fulminant and frequently fatal infection of the central nervous system (CNS) predominantly affecting children and young adults [1]. The organism is typically acquired through nasal exposure to contaminated warm freshwater during activities such as swimming or diving, from where it traverses the cribriform plate to invade the brain, causing rapid-onset inflammation, cerebral oedema, and necrosis [1,2,3].

Historically considered rare, PAM has demonstrated a rising global trend in reported cases, with increased geographic spread attributed in part to heightened diagnostic awareness and climate change-driven environmental shifts [4,5]. A previously published global review identified 381 cases between 1965 and 2018, with 41% of exposures occurring in the United States—mostly during summer months and in southern states—and an overall fatality rate of 92% [4]. Nevertheless, emerging data suggest that this pathogen’s ecological niche may be expanding, with recent reports from temperate regions [6,7,8]. Despite advances in molecular diagnostics—including polymerase chain reaction (PCR) and metagenomic next-generation sequencing (mNGS)—the diagnosis of PAM is often delayed due to its nonspecific early symptoms and rapid progression [9]. In the global case review, 36% of diagnoses occurred postmortem, and among survivors, early initiation of combination therapy was a critical factor [4]. Amphotericin B, often used with azoles, rifampicin, azithromycin, miltefosine, and corticosteroids, remains central to treatment; however, mortality is extremely high even with aggressive management [4,9].

Currently available data on *N. fowleri* PAM infections are mostly limited to case reports or small series, making it difficult to identify recent global trends in the disease pattern. There remains a critical need for a comprehensive, up-to-date analysis that consolidates global case data from the past 25 years to clarify evolving epidemiological patterns, diagnostic advances and practices, therapeutic strategies, and survival determinants. To address these gaps, this review synthesized contemporary evidence across paediatric and adult populations to inform improved clinical management of children and adults and targeted public health interventions.

## 2. Materials and Methods

### 2.1. Study Design

The present study retrospectively reviewed published cases of confirmed *N. fowleri* PAM infection for the period 2000 to 2024. The primary objective was to collect, synthesize and update adult and paediatric data on epidemiology, patient demographics, diagnosis, treatment, and clinical outcomes. The review was conducted according to the Preferred Reporting Items for Systematic Reviews and Meta-Analyses (PRISMA) 2020 guidelines (Supplementary S1) [10]. This review protocol is publicly available on PROSPERO (CRD420250652195), ensuring methodological transparency and adherence to systematic review standards.

### 2.2. Search Strategy

A comprehensive literature search was conducted using PubMed, Scopus, and the Cochrane Library. The search strategy employed combinations of Medical Subject Headings (MeSH) and free-text terms, including *“Naegleria fowleri”, “primary amoebic meningoencephalitis”, “central nervous system”, “p(a)ediatric”, “adult”, “treatment”, and “outcomes”* using Boolean operators *“AND”* and *“OR.”*

### 2.3. Screening and Data Extraction

Peer-reviewed case reports and case series published in English from January 2000 to January 2024, reporting confirmed cases of *N. fowleri* PAM infection with sufficient clinical detail, including patient demographics, diagnostic methods, treatment, and outcomes, were included. Two independent reviewers screened titles and abstracts; disagreements were resolved by a third reviewer. Data extracted included demographics, diagnosis, treatment and outcomes.

### 2.4. Statistical Analysis

Descriptive statistics—such as counts and percentages—were used to summarize categorical variables (e.g., sex, geographic region, diagnostic method, treatment type, and survival status). Where applicable, medians and interquartile ranges were reported for continuous variables (e.g., age, time to treatment, duration of hospitalization), depending on data availability. Patient ages were systematically reviewed and, where necessary, converted from months to years to ensure consistent classification. Individuals were categorized as pediatric if under 18 years of age and as adults if 18 years or older. Within each age group, descriptive analyses of survival and mortality were performed according to treatment subgroup (amphotericin B only, amphotericin B plus miltefosine, or other regimens). In addition, subgroup analyses were conducted to evaluate outcomes in pediatric and adult populations. Due to sample size limitations and reporting variability, these age-based comparisons were likewise interpreted descriptively rather than inferentially.

### 2.5. Ethical Considerations

As this study involved a review of previously published data from the scientific literature, ethical approval was not required. No individual patient-level data were collected beyond what was available in the public domain.

## 3. Results

### 3.1. Search Results

**Figure 1:**
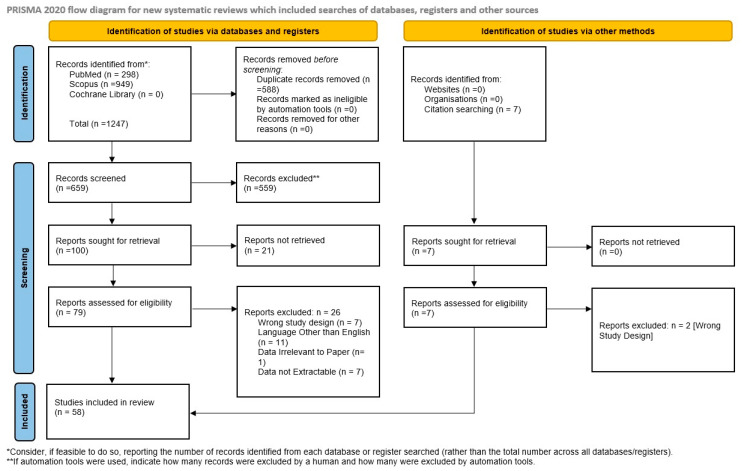
PRISMA flow diagram.

The search returned 1247 articles (PubMed: 298; Scopus: 949; Cochrane: 0). After removing duplicates, 659 unique records remained. Title screening led to the exclusion of 559 studies due to irrelevance to the topic. Abstract screening was performed on the remaining 100 records, and 21 studies were excluded due to inaccessibility or unavailable full texts. The remaining 79 full-text articles were evaluated and finally 58 studies, encompassing 66 individual patients, met all inclusion criteria and were included in the systematic review (Figure 1) [6,11,12,13,14,15,16,17,18,19,20,21,22,23,24,25,26,27,28,29,30,31,32,33,34,35,36,37,38,39,40,41,42,43,44,45,46,47,48,49,50,51,52,53,54,55,56,57,58,59,60,61,62,63,64,65,66,67].

### 3.2. Demographic Characteristics

Paediatric cases comprised the majority of the cohort with 37 cases (57%) with a median age of 8 years (IQR: 4–12), while 28 cases (43%) occurred in adults with a median age of 41 years (IQR: 28.75–51.25). Most patients were male (51/66, 77%), similar in paediatric and adult groups (Table 1 and Table 2).

### 3.3. Geographic Distribution and Temporal Trends

**Figure 2:**
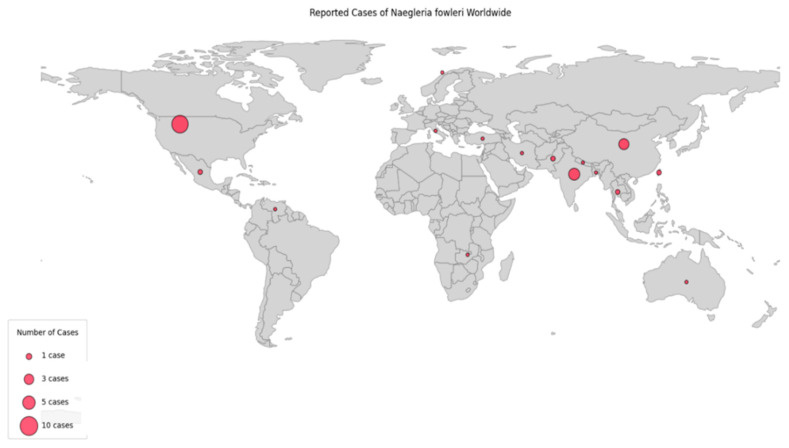
Global Distribution of *Naegleria fowleri* reported cases for the period 2000–2024.

Most of the published cases were concentrated in three primary regions. The United States accounted for 26 cases (40%), most notably in the southern states of Florida and Texas, each reporting six cases (9.2%), followed by Arkansas and Louisiana with three cases each (4.6%), California with two cases (3.1%) and Arizona, Georgia, Minnesota, Nebraska, New Jersey, Ohio, and North Carolina with 1 case each (1.5%). India contributed 12 cases (18.5%), with reports from regions such as Himachal Pradesh and Uttar Pradesh. From China, 10 cases (15.4%) were reported, primarily from Changsha, Fuzhou, and Zhejiang Province. Additional cases originated from Taiwan and Pakistan (two cases each, 3.1%). Single cases were reported from Australia, Bangladesh, Iran, Italy, Korea, Mexico, Nepal, Norway, Thailand, Turkey, Venezuela, and Zambia (each 1.5%) (Figure 2, Table 1 and Table 2).

**Figure 3:**
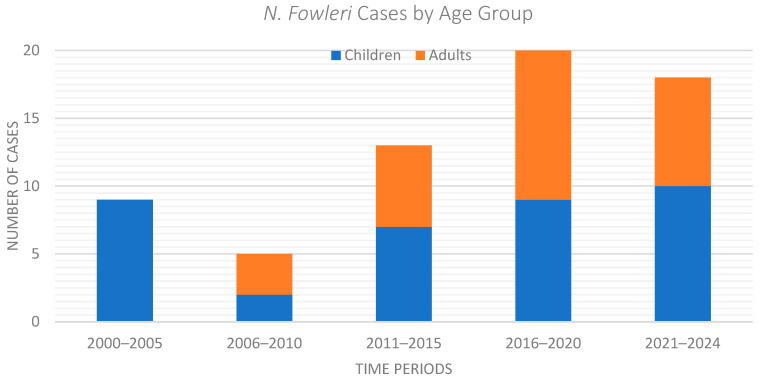
Temporal Distribution of *Naegleria fowleri* cases by Age Group.

An overall increase in the number of reported N. fowleri PAM cases was observed over the study period from 2000 to 2024. The average number of reported cases per year rose from approximately one case per year between 2000 and 2005 (5 cases), to nearly three cases per year between 2006 and 2019 (40 cases), and to just over four cases per year between 2020 and 2024 (21 cases). Notably, nearly one third of all reported cases (32.3%) have been documented since 2020, reflecting a recent rise in the published cases. The temporal distribution per age group is depicted in Figure 3.

### 3.4. Clinical Presentation and Diagnosis

The most reported initial symptoms included headache, fever, and altered mental status both in children and adults, whereby seizures were more frequently observed among pediatric patients. Diagnostic confirmation was typically achieved through cerebrospinal fluid (CSF) analysis, either by direct microscopy, staining, or PCR testing. Post-mortem diagnosis of *N. fowleri* PAM, by brain biopsy, was made in 18 cases (27.7%), including 13 children (35.1%) and 5 adults (17.9%) (Table 1 and Table 2).

### 3.5. Treatment and Management

Treatment approaches varied across the cases: combination antimicrobial therapy was administered in 57 cases (87.7%), including amphotericin B, miltefosine, fluconazole, azithromycin, rifampicin, and corticosteroids. Monotherapy, typically involving amphotericin B alone, was administered in only 2 cases (3%). Supportive measures included corticosteroids, mannitol for management of cerebral edema, and anticonvulsants in case of seizures. Due to rapid deterioration before the final diagnosis of *N. fowleri* PAM 6 patients (9.2%) did not receive any drugs for treatment. The overall median duration of treatment was 5 days (IQR: 3–13). The median treatment duration was longer (26 days, IQR: 21–28) among survivors compared to a median duration of 4 days (IQR: 2.75–6.5) among non-survivors, respectively.

Among pediatric patients, 17 were treated with amphotericin B without miltefosine, either alone or alongside other medications, 5 survived (29.4%), 11 died (64.7%), and 1 patient left against medical advice (5.9%). Ten children received amphotericin B combined with miltefosine, resulting in 3 survivors (30.0%) and 7 deaths (70.0%). Of the 10 pediatric patients treated with alternative antimicrobial regimens (excluding both amphotericin B and miltefosine), none survived.

In the adult cohort, 18 patients received amphotericin B-based therapy. Of these, 14 were treated with amphotericin B without miltefosine; 2 survived (14.3%) and 12 died (85.7%). Four adults received amphotericin B combined with miltefosine, of whom none survived. Also, the ten adults who were treated with alternative regimens (excluding both amphotericin B and miltefosine) died (Table 1 and Table 2).

Time from symptom onset to treatment initiation was reported for 11 of 65 cases overall. Among paediatric patients, onset-to-treatment timing was available for 9 of 37 cases, with a median time to treatment of 2 days (IQR: 1–3; range: 1–10). In the adult cohort, corresponding data were available for only 2 of 28 cases; treatment was initiated at 1 and 3 days after symptom onset (median: 2 days; IQR: 1.5–2.5).

### 3.6. Overall Survival

**Figure 4:**
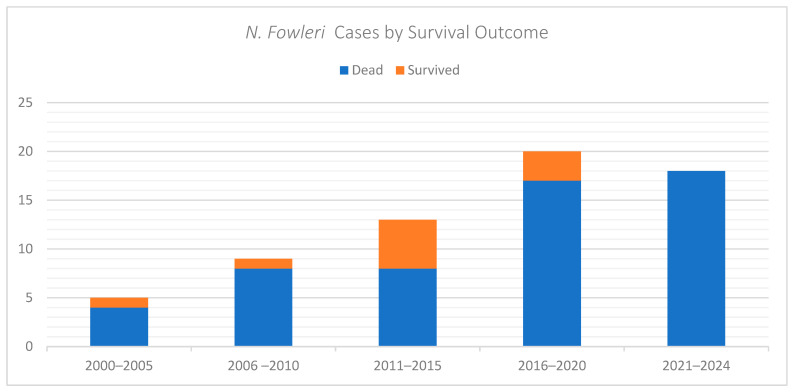
Temporal Distribution of *Naegleria fowleri* Cases by Survival Outcome.

The overall survival rate was 15.4% (10/65). All survivors received combination therapy involving multiple antimicrobial agents. Survival rate was highest in patients treated with amphotericin B with or without miltefosine, often administered within the first 24 to 48 h of symptom onset. Paediatric patients had a higher survival rate (8/37, 22%) compared to adults (2/ 28, 7.1%) (Table 1 and Table 2). The temporal distribution of *N. fowleri* PAM cases per outcome is depicted in Figure 4.

## 4. Discussion

This review presents an updated global synthesis of *N. fowleri* PAM cases, offering insights into temporal and demographic patterns and clinical outcomes observed over the past 24 years. Consistent with earlier reports, PAM remains a rare but devastating infection, with limited therapeutic success and a persistently high case fatality rate.

### 4.1. Mortality

Despite the incorporation of novel diagnostic technologies and intensified treatment strategies, mortality in our case series remained high at 83%. All survivors had received early, multi-drug therapy—typically initiated within 24–48 h of symptom onset—and regimens consistently included combination treatment with amphotericin B, miltefosine and other antimicrobial agents. In contrast, monotherapy yielded no survivors, and patients who received no targeted treatment, often due to rapid disease progression and early death, accounted for more than 10% of the cases. These findings reaffirm the narrow window for intervention in PAM and underscore the need for empirical treatment to be initiated promptly when the condition is suspected [4,5].

### 4.2. Survival

Our study revealed that pediatric cases comprised 57% of all reported infections, with a comparatively higher survival rate (22%) than adults (7.1%). While this mirrors previous findings [4], the reason for this discrepancy remains uncertain. Possible contributing factors include earlier healthcare access, heightened parental vigilance, and more aggressive interventions in pediatric intensive care units. Male predominance (78%) was noted in this series, aligning with previously reported epidemiological patterns [5]. This skew may reflect gendered behavioural exposure risks, such as increased participation in freshwater recreational activities, rather than intrinsic biological susceptibility. These findings seem to agree with Gharpure et al. who observed a similar predominance of pediatric cases and male patients, with exposure primarily linked to warm freshwater environments [3,4].

### 4.3. Temporal Increase

One notable trend observed in this review was the temporal increase in reported cases, particularly in the four years between 2020 and 2024, when over 22% of all cases were documented compared to 78% in the previous 20 years. This upward trend may partly be attributed to enhanced case detection through improved diagnostic modalities, including PCR and metagenomic sequencing. However, it also coincides with environmental changes such as increasing global temperatures and extended freshwater exposure periods, potentially expanding the ecological niche of and exposure to *N. fowleri* [6,67,68]. These findings highlight the need for cautious interpretation and reinforce the importance of establishing prospective, systematic reporting systems to detect early shifts in incidence and geographic range.

### 4.4. Climate Change

The role of climate change in the emergence and expansion of *N. fowleri* cannot be overlooked. As more water bodies reach optimal growth temperatures (25–40 °C) for the organism, the seasonal and geographic risk windows are widening [9,68,69]. This has been further exacerbated by anthropogenic activities, including urban encroachment on natural water systems, recreational water use, and poor water infrastructure in resource-limited settings. While strong ecological associations have been drawn between warming trends and pathogen spread, direct causality remains to be established, due to a lack of long-term environmental surveillance data. A more structured, global monitoring system integrating environmental sampling (e.g., water surveillance), case reporting, and geospatial analysis is urgently needed. To address these evolving challenges, the One Health approach presents a viable framework. Public awareness campaigns and clinician training should be strengthened to support early symptom recognition and prompt empirical therapy initiation. In addition, expanding access to advanced diagnostics like PCR and metagenomic sequencing at regional levels would significantly enhance diagnostic timeliness and accuracy, particularly in currently non-endemic or under-resourced regions.

### 4.5. Strengths and Weaknesses

This study’s strength lies in its comprehensive scope and structured data collection from multiple regions and decades, which enabled meaningful demographic and clinical subgroup analysis. It provides valuable initial data for public health officials and clinicians working on rare parasitic infections. Unlike previous reviews, our study distinctly stratified paediatric and adult cases, revealing higher survival rates in children and suggesting possible age-related differences in disease course or care access [70]. We also provided detailed subgroup analyses of treatment approaches, showing that early combination therapy—especially with amphotericin B and miltefosine—was linked to improved outcomes, particularly in paediatric patients. This adds critical age-specific insights to the existing literature, which has often emphasized early treatment without such demographic differentiation. However, several limitations must be acknowledged. The heterogeneity of the case reports, lack of standardized data across publications, and possible publication bias toward atypical or severe cases constrain broader generalizability. Furthermore, diagnostic disparities across regions and variable reporting standards complicate attempts to assess true incidence trends. The apparent increase in cases over time may reflect greater detection and awareness rather than a true increase in infection incidence. Similarly, the low number of reported cases from Africa may be influenced by underdiagnosis and underreporting, particularly in rural areas where access to medical care and diagnostic resources is more limited

In light of the consistently poor outcomes associated with PAM, prevention remains the cornerstone of public health strategies. Enhanced environmental surveillance—particularly in warm, recreational freshwater settings—should be prioritized during peak summer months. An additional low-cost preventive strategy is the use of nose clips during swimming or freshwater recreational activities in N. fowleri-endemic areas, which may reduce nasal exposure to contaminated water. Combined with real-time public health advisories and heightened clinical vigilance, such measures could facilitate earlier intervention and reduce mortality.

## 5. Conclusions

*Naegleria fowleri* PAM remains highly lethal with only modest improvement over the years, despite advancements in diagnostics and combination therapies. While paediatric patients may show better survival under aggressive management, the overall prognosis remains dismal for infected individuals. The increasing number of reported cases in recent years, alongside a broader geographic spread, underscores the potential influence of climate change on the organism’s niche. There is an urgent need for coordinated global surveillance, enhanced diagnostic capacity, and awareness campaigns to support early detection and improve outcomes. As environmental changes continue to shape the landscape of emerging infectious diseases, proactive and collaborative responses will be essential to mitigate the threat posed by this rare but deadly pathogen.

## Figures and Tables

**Table 1:** Characteristics of published adult *Naegleria fowleri* PAM cases, period 2000–2024.

Author	Year	Region, Country	Sex	Age	Initial Symptoms	Treatment (Drug A − Dose + Drug B − Dose)	Method of Diagnosis	Location of Infection	Duration of Treatment (days)	Initiation of Treatment Relative to First Onset of Symptoms	Outcome
Wei HY et al. [11]	2024	Taiwan	F	30	headache, neck and shoulder stiffness	N/A	qRT PCR	N/A	N/A	N/A	Death
Hong et al. [15].	2023	Korea	M	52	headache, fever	Amphotericin B + Fluconazole + Azithromycin + Rifampicin	CSF PCR	N/A	13	N/A	Death
Wang et al. [30]	2023	Zhousan Island, China	M	62	vomiting, headache, behavioral change, coma, fever and chills	N/A [Empirical Antibiotics for Meningitis]	CSF Macro—Genome Sequencing (post-mortem)	N/A	3	3 Days	Death
Wu et al. [35]	2023	Zhejiang Province, China	M	42	fever	Meropenem (2000 mg every 8 h) + Metronidazole (500 mg every 8 h) + Fluconazole (800 mg everyday) + mannitol + Norepinephrine (16–24 μg/min	CSF metagenomic next-generation sequencing (mNGS)	N/A	2	N/A	Death
Puthanpurayil et al. [38]	2023	India	M	36	headache, photophobia, nausea, fever, generalized tonic–clonic seazures	Ceftriaxone + Acyclovir + Dexamethasone	CSF Wet Mount Light Microscopy + CSF culture	Nostrils	N/A	N/A	Death
Soontrapa et al. [17]	2022	Saraburi Province, Thailand	F	40	headache, fever	Amphotericin B + Dexamethasone + Fluconazole + Azithromycin + Rifampicin	CSF PCR	Nostrils	5	N/A	Death
Chen et al. [56]	2022	Fuzhou, China	M	47	fever, weakness, backache	Meropenem (6 g/d) + Vancomycin (2 g/d) + Dexamethasone (10 mg/d)	CSF metagenomic next-generation sequencing (mNGS)	N/A	4	N/A	Death
Harris et al. [65]	2021	California, USA	M	40	fever, sore throat, cough, lethargy	Ceftriaxone + Ampicillin + Amphotericin B + Rifampicin + Fluconazole + Azithromycin + Miltefosine	CSF Giemsa Stain + real-time PCR	N/A	5	N/A	Death
Hamaty et al. [57]	2020	New Jersey, USA	M	29	fever, altered levels of consciousness	Dexamethasone (10 mg) + Vancomycin + Meropenem + Acyclovir + Doxycycline + Azithromycin + Atovaquone + Amphotericin B + Fluconazole	CSF Smear	N/A	3	N/A	Death
Ganesan et al. [63]	2020	Tamil Nadu, India	M	47	headache, vomiting, altered sensorium	Amphotericin B (150 mg) + Azithromycin	CSF Wet Mount Light Microscopy	N/A	7	N/A	Survived
Che et al. [39]	2019	Guangdong Province, China	M	38	fever, headache, vomiting, altered mental status	Penicillin + Ceftriaxone	CSF metagenomic next-generation sequencing (mNGS)	N/A	3	N/A	Death
Mushtaq et al. [40]	2019	Pakistan	M	44	fever, headache, generalized weakness	Amphotericin B + Dexamethasone + Fluconazole + Rifampicin + Miltefosine + Levetirecetam	CSF Wet Mount Light Microscopy + CSF PCR	N/A	4	N/A	Death
Cope et al. [47]	2018	North Carolina, USA	F	18	headache, fever, lethargy	Amphotericin B + Fluconazole + Azithromycin + Rifampicin + Miltefosine − 1 dose	CSF PCR	Nostrils	N/A	N/A	Death
Wang et al. [36]	2018	Zhejiang Province, China	M	42	headache, fever	Meropenem + Linezolid + Dexamethasone + Amphotericin B (50 mg/day) + Fluconazole (0.4 g/day)	CSF metagenomic next-generation sequencing (mNGS) + CSF PCR	N/A	11	N/A	Death
Baral et al. [58]	2018	Nepal	M	74	fever, headache, aphasia	AmphotericinB + Flucytosine + Voriconazole + Azithromycin + Rifampicin + Miltefosine	Brain Biopsy	N/A	10	N/A	Death
Chomba et al. [21]	2017	Lusaka, Zambia	M	24	seizures, fever	Amphotericin B (50 mg)	CSF Wet Mount Light Microscopy	N/A	1	N/A	Death
McLaughlin et al. [41]	2017	Queensland, Australia	M	56	headache, photophobia, nausea, fever, vomiting, neck stiffness	Amphotericin (1.5 mg daily) + Amphotericin (50 mg every 12 h) + Rifampicin (600 mg daily) + Azithromycin (500 mg daily) + Fluconazole (800 mg daily) + Dexamethasone (8 mg twice daily) + chlorpromazine (50 mg every 4 h)	CSF Wet Mount Light Microscopy + CSF PCR	N/A	3	N/A	Death
Chen et al. [42]	2016	Hangzhou, China	M	43	headache, fever, myalgia, fatigue	Amphotericin B (5 mg initial dose followed by 10 mg on day 2, 25 mg on day 3, and 50 mg/day thereafter) + Fluconazole (400 mg/day)	CSF Wet Mount Light Microscopy + CSF PCR	N/A	9	N/A	Death
Stubhaug et al. [45]	2016	Oslo, Norway	F	71	nausea, fever, fatigue, vomiting	Meropenem + Vancomycin	Brain Autopsy (Post-Mortem)	N/A	3	N/A	Death
Yoder et al. [14]	2012	Louisiana, USA	M	28	headache, neck stiffness, back pain, vomiting	Dexamethasone + Ceftriaxone + Linezolid + Acyclovir + Amphotericin B + Rifampicin	CSF Light Microscopy + CSF Cytospin + CSF PCR	Nostrils	3	N/A	Death
	2012	Louisiana, USA	F	51	altered mental status, nausea, vomiting, poor appetite, listlessness, fatigue, fever	N/A	Brain Biopsy (post-mortem/autopsy)	N/A	N/A	N/A	Death
Mei-Yu et al. [28]	2012	Taiwan	M	75	headache, fever, right arm myoclonic seizures	Amphotericin B (50 mg q.d.)	CSF Wet Mount Light Microscopy + CSF Cytospin + CSF PCR	N/A	21	N/A	Death
Midha et al. [43]	2012	India	M	73	fever, neck pain, seizures, altered sensorium	Amphotericin B (1 mg/kg/day) + Rifampicin (600 mg) + Ceftriaxone + Vancomycin	CSF Giemsa Stain + CSF Culture	N/A	30	N/A	Survived
Tuppeny [26]	2011	Florida, USA	M	22	headache, neck pain, photosensitivity	Vancomycin + Ceftriaxone + Amphotericin B + Fluconazole + Azithromycin + Rifampicin	CSF Wet Mount Light Microscopy	N/A	N/A	1 Day	Death
Sharma et al. [59]	2011	Himachal Pradesh, India	F	21	fever, headache, vomiting, seizures	Ceftriaxone + Vancomycin + Acyclovir + Steroid + Amphotericin	CSF Wet Mount Light Microscopy	N/A	5	N/A	Death
Gupta et al. [52]	2009	India	M	20	fever, headache, loss of vision, hearing loss, slurred speech, difficulty in swallowing, urine retention	Ceftriaxone + Amikacin + Mannitol + Amphotericin B (1.5 mg/kg) + Rifampicin (450 mg)	CSF Culture	N/A	16	N/A	Death
Shakoor et al. [27]	2008	Karachi, Pakistan	M	30	headache, fever, seizures	Amphotericin B + Fluconazole + Rifampicin	CSF Wet Mount Light Microscopy	N/A	4	N/A	Death
Matthews et al. [50]	2008	Texas, USA	M	22	photosensitivity, altered mental status, headache	N/A	Brain Autopsy (post-mortem)	Ruptured Eardrum	4	N/A	Death

**Table 2:** Summary of published pediatric PAM cases period 2000–2024.

Author	Year	Region, Country	Sex	Age	Initial Symptoms	Treatment (Drug A − Dose + Drug B − Dose)	Method of Diagnosis	Location of Infection	Duration of Treatment (Day/s)	Initiation of Treatment Relative to First Onset of Symptoms (Days)	Outcome
Lin et al. [37]	2024	China	F	6	headache, fever, vomiting, lethargy	Meropenem + Vancomycin + Acyclovir + Epinephrine + Dobutamine + Mannitol + Immunoglobulin Infusion + Amphotericin B + Rifampicin	CSF metagenomic next-generation sequencing (mNGS)	N/A	2	N/A	Death
Song et al. [66]	2024	China	M	14	fever, headache, seizures, altered consciousness	Amphotericin B (10 mg/dose) + Rifampicin (0.3 g/dose) + Sulfamethoxazole (400 mg)-Trimethoprim (80 mg) + Fluconazole (0.4 g/dose) + Artesunate-Pyronaridine (80 mg/dose)	CSF metagenomic next-generation sequencing (mNGS)	N/A	45	N/A	Death
Hebbar et al. [31]	2005	India	M	0.5	fever, headache, seizures, altered consciousness	Amphotericin B + Chloramphenicol + Metronidazole	CSF Giemsa Stain	N/A	1	3	Death
Rai et al. [51]	2008	Indian	M	0.7	fever, vomiting, altered consciousness	Amphotericin B (1.5 mg/kg/day) + Chloramphenicol (100 mg/kg/day) + Rifampicin	India Ink presentation	N/A	21	4	Survived
Vargas-Zepeda et al. [46]	2005	Sonora, Mexico	M	10	severe headache, vomiting, fever	Dexamethasone + Ceftriaxone + Rifampicin + Amphotericin B + Fluconazole	CSF Wet Mount Light Microscopy	N/A	30	1	Survived
Maloney et al. [20]	2023	Nebraska, USA	M	8	altered mental status, headache, malaise, fatigue, fever, facial rash	Amphotericin B + Fluconazole + Azithromycin + Rifampicin + Miltefosine	CSF Giemsa Stain	N/A	1	N/A	Death
Eger et al. [32]	2023	Texas, USA	M	3	fever, poor oral intake, vomiting, somnolence, nasal congestion	Amphotericin B + Dexamethasone + Fluconazole + Azithromycin + Rifampicin + Miltefosine	CSF Giemsa Stain + CSF PCR	Nostrils	8	1	Death
McCormick-Baw et al. [33]	2023	Texas, USA	M	8	fever, headache, altered mental status	Amphotericin B + Dexamethasone + Fluconazole + Azithromycin + Rifampicin + Miltefosine	CSF Giemsa Stain + CSF PCR	N/A	6	N/A	Death
Puthanpurayil et al. [38]	2023	Palakkad, India	M	4	altered sensorium, seizures, fever, nasal discharge, headache, vomiting	Ceftriaxone + Vancomycin + Dexamethasone + Amphotericin B + Fluconazole + Cotrimoxazole + Rifampicin	CSF Wet Mount Light Microscopy + pan-FLA PCR (post-mortem)	N/A	N/A	N/A	Death
Brener et al. [54]	2023	N/A	F	17	fever, headache, sore throat, ear pain, dizziness	Metronidazole + Vancomycin + Ceftriaxone + Acyclovir + Doxycycline.	CSF PCR (post-mortem)	N/A	11	N/A	Death
Zhou et al. [55]	2022	Changsha, China	M	9	fever, vomiting	Ibuprofen + Piperacillin/Tazobactam + Ceftriaxone + Vancomycin + Mannitol + Methylprednisolone + Human Immunoglobulin	CSF metagenomic next-generation sequencing (mNGS) (post—mortem)	N/A	19	N/A	Death
Huang et al. [19]	2021	China	M	8	headache, vomiting, fever	Meropenem + Vancomycin + Ceftriaxone	CSF PCR (post mortem)	N/A	25	1	Death
Anjum et al. [64]	2021	Florida, USA	M	13	headache, fever, vomiting	Ceftriaxone + Acyclovir + Vancomycin + Mannitol + Miltefosine + Amphotericin B + Fluconazole + Rifampicin + Azithromycin + Dexamethasone		N/A	4	N/A	Death
Celik et al. [22]	2020	Mersin, Turkey	M	0.03	irritability, inability to suck, fever	Amphotericin B + Dexamethasone + Fluconazole + Azithromycin + Rifampicin	CSF PCR	Nostrils	N/A	2	Death
Sazzad et al. [13]	2019	Nilphamari District, Bangladesh	M	15	headache, fever	Ceftriaxone + Metronidazole + Meropenem + Gentamicin	CSF PCR (post mortem)	N/A	6	N/A	Death
Vareechon et al. [62]	2019	California, USA	M	8	headache, neck-stiffness, photophobia, vomiting, seizures, delirium	Vancomycin + Ceftriaxone + Acyclovir + Dexamethasone + Lorazepam + Fluconazole + Amphotericin B + Azithromycin + Posaconazole + Rifampicin + Miltefosine	CSF Giemsa Stain	N/A	3	N/A	Death
Mittal et al. [23]	2018	Haryana, India	F	0.66	fever, rigors, chills, abnormal body movements, vomiting, generalized tonic–clonic seizures, decreased oral acceptance and decreased urine output	Ceftriaxone + Vancomycin + Amphotericin B + Acyclovir	CSF PCR	N/A	N/A	2	N/A [Left Against Medical Advice]
Heggie et al. [12]	2017	Arkansas, USA	F	12	headache, vomiting, fever	Amphotericin B + Fluconazole + Azithromycin + Rifampicin + Dexamethasone + Miltefosine	CSF PCR	N/A	30	N/A	Survived
Stowe et al. [25]	2017	Texas, USA	M	4	fever, altered mental status, seizure, headaches, vomiting, difficulty ambulating	Amphotericin B + Dexamethasone + Fluconazole + Azithromycin + Rifampicin + Miltefosine	CSF Wet Mount Light Microscopy + CSF PCR (post-mortem)	N/A	2	N/A	Death
	2017	Texas, USA	M	14	headache, generalized muscle weakness, fever, vomiting, confusion	Vancomycin + Ceftriaxone + Amphotericin B + Fluconazole + Azithromycin + Rifampicin + Miltefosine	CSF PCR (post-mortem)	N/A	4	N/A	Death
Wagner et al. [53]	2017	Monagas, Venezuela	M	0.33	N/A	N/A	CSF DNA Extraction + PCR (post-mortem)	N/A		N/A	Death
Dunn et al. [18]	2016	Arkansas, USA	F	12	headache, lethargy, fever, nausea, vomiting	Amphotericin B + Dexamethasone + Fluconazole + Azithromycin + Rifampicin + Miltefosine	CSF Gram Stain + CSF Cytospin	N/A	27	2	Survived
Linam et al. [24]	2015	Arkansas, USA	F	12	headache, fever, nausea, vomiting, somnolence	Amphotericin B (1.5 mg/kg/day) + Dexamethasone + Fluconazole (10 mg/kg/day) + Azithromycin (10 mg/kg/day) + Rifampicin (10 mg/kg/day) + Miltefosine (50 mg/day)	CSF Giemsa Stain	N/A	26	N/A	Survived
Chauhan et al. [16]	2014	Himachal Pradesh, India	M	6	headache, fever, altered sensorium	Amphotericin B (1 mg/kg) + Fluconazole (8 mg/kg) + Rifampicin (10 mg/kg)	CSF Wet Mount Light Microscopy + CSF culture	N/A	21	N/A	Survived
Cope et al. [34]	2013	Louisiana, USA	M	4	diarrhea, vomiting, poor oral intake, headache, fever	Vancomycin + Ceftriaxone + Piperacillin/Tazobactam + Acyclovir	Brain Biopsy (postmortem/autopsy)	N/A	5	N/A	Death
Lopez et al. [44]	2012	Florida, USA	M	13	headache, nuchal rigidity, fever, photophobia	Ceftriaxone (100 mg/kg divided every 12 h) + Vancomycin (60 mg/kg divided every 6 h) + Lorazepam (0.1 mg/kg intravenously) + Fosphenytoin (loading dose 20 mg/kg) + Amphotericin B	Brain Biopsy (post mortem/autopsy)	N/A	N/A	N/A	Death
Movahedi et al. [60]	2012	Iran	M	0.41	fever, eye gaze	Ceftriaxone (100 mg/kg/day) + Vancomycin (15 mg/kg/dose) + Rifampicin (10 mg/kg) + Amphotericin B (1 mg/kg/day)	CSF Wet Mount Light Microscopy + CSF Culture	N/A	N/A	N/A	Survived
Kemble et al. [7]	2011	Minnesota, USA	F	7	headache, abdominal pain, neck pain	Lorazepam + Ceftriaxone + Vancomycin	CSF PCR	Right Frontal Lobe	N/A	N/A	Death
Yadav et al. [29]	2011	Uttar Pradesh, India	M	0.10	fever, multi-focal seizures	Amphotericin B + Fluconazole + Rifampicin	CSF Wet Mount Light Microscopy + CSF Culture	Nostrils	28	10	Survived
Matthews et al. [50]	2008	Arizona, USA	M	14	headache, stiff neck, fever			N/A	1	N/A	Death
Matthews et al. [50]	2008	Florida, USA	M	14	ear pressure, headache, vomiting		Brain Autopsy (Post-Mortem)	N/A	1	N/A	Death
Matthews et al. [50]	2008	Florida, USA	M	11	headache, fever, nausea, vomiting, confusion	Amphotericin B + Epinephrine + Mannitol + Fluconazole + Ceftriaxone + Azithromycin + Rifampicin	CSF Light Microscopy	N/A	2	N/A	Death
Matthews et al. [50]	2008	Florida, USA	M	10	headaches, body aches, high fever, nausea, vomiting, fainting	Amphotericin B + Rifampicin + Azithromycin + Fluconazole	CSF Light Microscopy	N/A	2	N/A	Death
Matthews et al. [50]	2008	Texas, USA	M	12	fever, disoriented, lethargic	Amphotericin B + Rifampicin + Azithromycin	CSF Light Microscopy	N/A	5	N/A	Death
Cogo et al. [61]	2004	Este, Italy	M	9	fever, headache	Ceftriaxone + Corticosteroids + Acyclovir + Mannitol	Brain Autopsy (postmortem)	N/A	6	N/A	Death
Centers for Disease Control and Prevention (CDC) [49]	2003	Georgia, USA	M	11	headache, vomiting, fever, lethargy	Amphotericin + Rifampicin + Ketoconazole.	Brain Autopsy (post-m ortem)	N/A	4	N/A	Death
Shenoy et al. [48]	2002	Mangalore, India	N/A	0.41	fever, vomiting, convulsions	Amphotericin B (0.6 mg/kg) + Ceftriazone (100 mg/kg)	CSF Culture	Nostrils	2		Death

## Data Availability

No new data were created or analyzed in this study. Data sharing is not applicable to this article.

## Supplementary Materials

The following supporting information can be downloaded at: https://www.mdpi.com/article/10.3390/idr18020025/s1. Supplementary S1: PRISMA 2020 Checklist
